# Walter Michael Kuehl, MD - October 25, 1939 - April 30, 2023

**DOI:** 10.46989/001c.87881

**Published:** 2023-09-19

**Authors:** Leif Bergsagel

**Affiliations:** 1 Department of Medicine, Mayo Clinic Arizona, Scottsdale, AZ, USA

**Figure:**
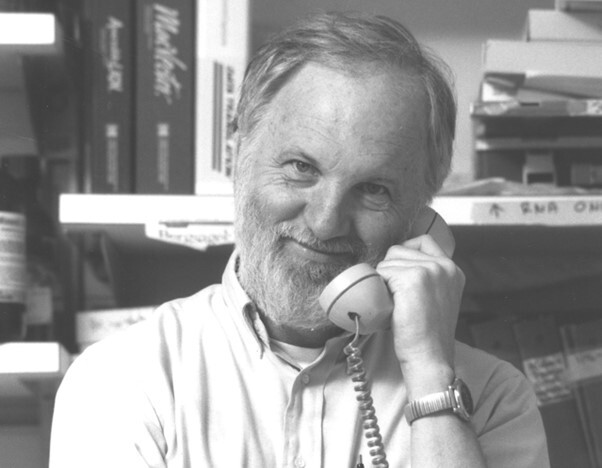


Mike, a long-time member of the NIH community, passed away peacefully on April 30, 2023, after a courageous 12-year batter with renal cell carcinoma. Following graduation from Harvard Medical School, Mike first started working at the NHLBI in the early seventies with Dr. Robert Adelstein studying myosin.^1^ He went on to post-graduate studies with Dr. Matthew Scharff at Albert Einstein College of Medicine studying immunoglobulin production^2^ and the effects of somatic cell hybridization in mouse myeloma lines.^3^ It was during this time (1974) that Mike published several of the very first papers characterizing monoclonal antibodies. The work with Dr. Scharff spurred his long-term interest in plasma cell biology that continued throughout his career. He joined the faculty of the University of Virginia in 1975 where he focused on the regulation of immunoglobulin production in B cell development.^4^ In 1980 he returned to the NIH, where he learned molecular genetics in Dr. Phil Leder’s lab.^5^ He was then recruited by Dr. John Minna to develop a new molecular genetics unit at the NCI/Navy Medical Oncology Branch in 1982. He initially studied the role of MYB in hematopoietic differentiation,^6^ until he turned his attention to understanding the genetics of human multiple myeloma in the mid-nineties.

Together with Dr. Leif Bergsagel, a former postdoctoral fellow and longtime collaborator, Mike identified recurrent immunoglobulin heavy chain gene translocations that form the basis for the molecular classification of myeloma and are routinely assayed in patients’ samples today.^7^ He also identified the central role of MYC^8^ and NFKB^9^ in myeloma progression. It is hard to overstate the importance of these accomplishments in the myeloma field, for which Mike received the prestigious Waldenstrom Award from the International Myeloma Workshop in 2011. Following retirement, Mike continued to be involved in science as an NCI emeritus investigator, reviewing manuscripts, writing review articles, and participating in Dr. Javed Khan’s lab meetings.

With a twinkle in his eyes, a quick smile, hearty laugh, and a joyous enthusiasm for science, Mike left an indelible mark on the people fortunate enough to train in his laboratory

-Source

**Figure:**
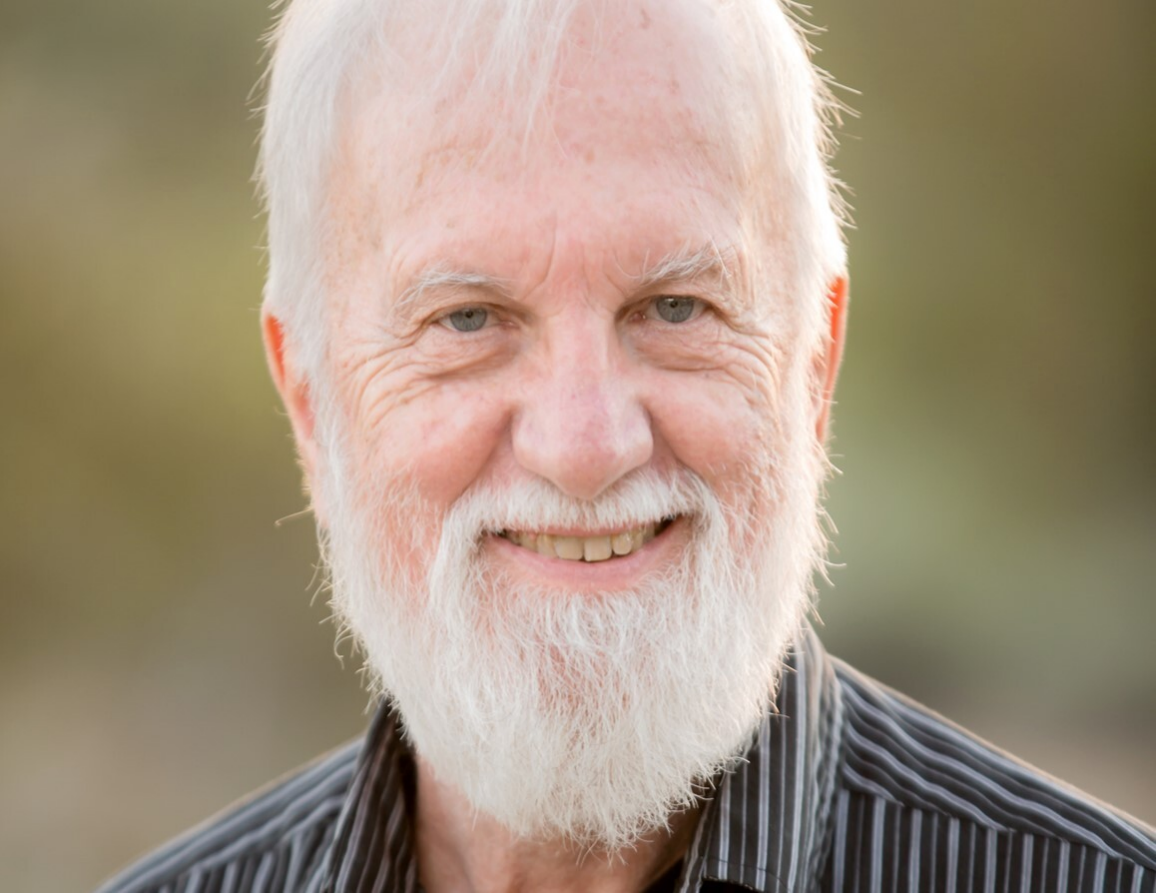


Mike freely shared his intellectual insight and unique resources with numerous researchers, contributing to countless studies to understand the signaling networks involved in pathogenesis and drug response in multiple myeloma. He was an inspiring teacher and mentor. With a twinkle in his eyes, a quick smile, hearty laugh, and a joyous enthusiasm for science, Mike left an indelible mark on the people fortunate enough to train in his laboratory including Drs. Ethan Dimitrovsky, Tim Bender, Leif Bergsagel, Marta Chesi, Rafael Fonseca, and Adriana Zingone, as well as his long-time assistant Leslie Brents. For the people that knew him what was most striking was his intellectual acumen, insatiable curiosity, and rigor in approaching a scientific question. As a result, many of his manuscripts were accepted without substantial revision. He maintained an encyclopedic knowledge of all aspects of plasma cell development and often served as a point of reference for others in the field when trying to get to the bottom of a complex scientific question. He was a very helpful and thorough reviewer and took upon himself to always improve, not simply criticize, the submitted works. He was a singular role model for a scientific researcher, always pushing to balance work and family life.

“Don’t postpone joy”

Mike was a remarkable athlete, playing squash well into his late seventies, and taking a cross-country bike ride with his daughter Sarah while in his late sixties. Mike was loved, respected, and admired by his lab members, perhaps highlighted by the fact that he was best man at the wedding of two former post-doctoral fellows, Drs. Leif Bergsagel and Marta Chesi. Mike’s motto was “Don’t postpone joy”, and he never did. We will all miss him greatly.

Written by Leif Bergsagel, with editorial input from Peter Aplan, incorporating comments from David Margulies, Leslie Brents, Lanny Kirsch, Konrad Huppi, Beverly Mock and Javed Khan.
